# Broader use of hepatitis B virus vaccine: Efficacy in those who lost hepatitis B surface antigen during follow-up

**Published:** 2011-06-01

**Authors:** Rakesh Kumar, Babita Agrawal

**Affiliations:** 1 Department of Laboratory Medicine and Pathology, University of Alberta, Edmonton, AB, Canada; 2 Department of Surgery, University of Alberta, Edmonton, AB, Canada

**Keywords:** Hepatitis B vaccine, Hepatitis B surface antigen, Liver fibrosis

Dear Editor,

The article "efficacy of hepatitis B vaccine in those who lost hepatitis B surface antigen during follow-up" published recently in Hepatitis Monthly [1], described the efficacy of HBV vaccine in chronic HBV patients who had been found negative for HBsAg and HBV DNA in sera. The manuscript was highlighted by an Editorial in the same issue [2]. The manuscript dealt with a group of chronic HBV patients who were described as having occult HBV [3], with one major difference; usually occult HCV is defined as the presence of low levels of HBV viremia in the absence of detectable HBsAg but detectable anti-HBcAb, whereas in this manuscript [1] the authors considered patients with undetectable HBsAg (with prior positive HBsAg) as well as undetectable HBV DNA levels (< 50 copies/mL of HBV DNA) with anti-HBe and anti-HBc antibodies. Thirty-four such patients and 52 control healthy individuals were vaccinated with three doses of HBV vaccine (Engerix-B, SmithKline Beecham); the antibody levels were determined one month after the last dose of vaccination. In the control group, 87% of individuals developed protective levels of anti-HBs antibodies as has been reported earlier [4]. The major finding of the manuscript was that in 24% of the selected occult HBV patients, protective levels of anti-HBs antibodies developed. However, the anti-HBs antibody levels were significantly lower than those of the healthy individuals (mean ± SD: 68 ± 32.66 vs. 344.6 ± 38.99, p < 0.001). The authors suggested that "the development of anti-HBs antibody may be a clue showing that they have protection and are less likely prone to develop chronic hepatitis, cirrhosis and hepatocellular carcinoma". This is an interesting study as it deals with HBV in patients whose disease outcome is not very clear. Also the study suggests that the role of HBV vaccine could be extended from prevention to therapeutic in certain cases of chronic HBV. However, the sole appearance of anti-HBs antibodies cannot be taken to reflect viral clearance or non-progression to liver diseases. It has been shown recently that seroconversion from HBsAg positive to anti-HBs antibodies positive does not necessarily correlate to viral clearance [5]. In this respect, it is important to note that perhaps in patients with occult HBV and undetectable serum HBsAg, there may be no or reduced tolerance against HBsAg as compared to those with active chronic HBV with high serum levels of HBsAg and therefore, the current HBV vaccine may be more effective in inducing anti-HBs antibodies. However, despite the presence of anti-HBs antibodies, low levels of viral replication may continue in the hepatocytes leading to further liver diseases such as cirrhosis, fibrosis and hepatocellular carcinoma. It is imperative that the patients with positive response to HBV vaccine in the current study [1], be followed for a longer duration to clearly examine the role of the induced anti-HBs antibodies in viral clearance or therapy of occult chronic HBV. As acknowledged by the authors [1], liver biopsies could have provided more information regarding viral clearance in response to the induction of anti-HBs antibodies. In addition, the study needs to be expanded to a larger number of patients to make important conclusions. Nevertheless, the paper represents an important study in examining the role of the current HBV vaccine in inducing protective anti-HBs antibodies in patients with occult HBV and may prove to be useful in expanding the role of the current HBV vaccine in therapy of occult chronic HBV.
